# The effectiveness of the comprehensive corrective exercise program on kinematics and strength of lower extremities in males with dynamic knee valgus: a parallel-group randomized wait-list controlled trial

**DOI:** 10.1186/s12891-022-05652-8

**Published:** 2022-07-22

**Authors:** Esmaeil Mozafaripour, Foad Seidi, Hooman Minoonejad, Mohammad Bayattork, Fatemeh Khoshroo

**Affiliations:** 1 Department of Health and Sports Medicine, Faculty of Physical Education and Sport Sciences, University of Tehran, North Karegar St, P.O.B:1439813117, Tehran, Iran; 2 Department of Sport Sciences and Physical Education, Faculty of Humanities Science, University of Hormozgan, Bandar Abbas, Iran

**Keywords:** Dynamic knee Valgus, Comprehensive corrective exercise program, Biomechanics, Muscle strength dynamometer

## Abstract

**Background:**

Dynamic knee valgus (DKV) is a prevalent movement impairment widely regarded as a risk factor for lower extremity disorders such as patellofemoral pain syndrome. The present study aimed to investigate the effectiveness of the comprehensive corrective exercise program (CCEP) on kinematics and strength of lower extremities in males with DKV.

**Methods:**

Thirty asymptomatic young men with DKV between the ages of 18 and 28 years participated in this study. They were randomly assigned to the intervention (*n* = 15) and control groups (*n* = 15). The intervention group performed the CCEP for three sessions per week for eight weeks, while the control group only did activities of daily living. Hip external rotator and abductor muscle strength and three-dimensional lower extremity kinematics consisting of knee varus/valgus, femur adduction/abduction, femur medial/lateral rotation, and tibial medial/lateral rotation were measured at the baseline and post-test. The data were analyzed using the analysis of covariance (ANCOVA).

**Results:**

There were significant improvements in all kinematics variables in the intervention group after the 8-week CCEP. Moreover, the strength of abductor and external rotator muscle improved in the intervention group (*P* < 0.05).

**Conclusions:**

The CCEP led to substantial improvements in the selected variables of lower extremity kinematics and muscle strength in participants with DKV during a single-leg squat. These results imply that practitioners should adopt a comprehensive approach to pay simultaneous attention to both proximal and distal segments for improving DKV.

**Trial registration:**

The protocol has been approved in the Registry of Clinical Trials (Registration N: IRCT20180821040843N1) on 2018-12-30.

## Highlights

The CCEP can improve lower extremity kinematics and muscle strength in people with DKV.

Proximal and distal segments should be considered simultaneously for improving DKV.

The CCEP is recommended to improve DKV.

## Introduction

Dynamic knee valgus (DKV) is a common movement impairment characterized by the combination of tibial and femoral rotation resulting from distal and proximal segments [1–4]. It is attributed to multi-joint and multi-plane compensations that cause varying degrees of increase in joint kinematics variables, including hip adduction and internal rotation as well as knee abduction and internal rotation [1, 4]. These excessive motions in frontal and transverse planes during functional activities, such as walking and running, are associated with the increased risk of lower extremity musculoskeletal disorders such as patellofemoral pain syndrome (PFPS) [5–8] and acute sports injuries such as anterior cruciate ligament rupture [9, 10]. These disorders and injuries can result in significantly adverse consequences. Therefore, it is necessary for researchers and clinicians to identify the possible causes and contributing factors to DKV so that they can prevent such injuries [11].

In this regard, some researchers have attempted to design appropriate exercise programs for reducing DKV, mainly formulated based on structural or functional approaches [12]. In the structural approach, changes in impaired movement patterns and malalignments are mainly attributed to peripheral factors assumed to lead to compensatory adjustments in the strength and length of involved muscles [13, 14]. This approach focuses merely on strengthening weakened muscles while ignoring other related compensatory changes [13]. Many researchers have investigated the effectiveness of the related exercise programs on DKV in dynamic tasks, focusing on hip stabilizers based on the supposition that weakness in hip external rotators and abductors can cause excessive hip internal rotation and adduction during load-bearing posture, and thus a larger valgus angle [15–19]. Interestingly, these studies have reported conflicting results. Besides, most of them have not confirmed the efficacy of the intervention programs [11, 20, 21]. Palmer et al. (2015) studied the effectiveness of an isolated hip abductor strengthening program on knee kinematics. The authors reported no statistically significant reductions in DKV and hip internal rotation [11]. On the contrary, Brindle et al. (2003) have verified the effectiveness of the strengthening exercise program targeting hip abductors and external rotators to reduce hip internal rotation and thus DKV [22].

On the other hand, the functional (neurological) approach is based on the premise that the interaction between different system elements provides valuable information about the overall performance and system behavior for generating and controlling motion [23, 24]. According to this approach, although it is important to pay adequate attention to muscle strength and length in impaired movement patterns such as DKV, the main focus needs to be on neuromuscular factors such as motor control due to the belief that changes in motor unit recruitment will ultimately alter motor programming [25, 26]. However, these concepts are not necessarily operationalized beyond the site of problem which is likely to have a cascading effect throughout all other segments.

Aligned with functional approach, Seidi and colleagues (2014) have evolved the comprehensive approach aiming to achieve the best intervention outcome possible by attending strong points of previous approaches and obviating their shortcomings [24]. Likewise, it has been designed based upon the interaction between the muscular, articular, and neural subsystems for generating movement [23, 26]. The main contribution of this approach is to comprehensively and integratively consider muscle activation, movement pattern, and alignment of all segments simultaneously across the whole body rather than merely the single site of the problem during both assessment phase and training protocol design. Moreover, to design the intervention, due attention should be paid not only to the parametric abilities such as muscle length and strength, but also to neuromuscular factors and compensatory changes over the distal and proximal segments [27]. Thus far, the efficacy of this approach has been investigated only for upper quarter malalignments such as upper crossed syndrome [27]. To the best of our knowledge, no study has ever dealt with DKV considering all three components of alignment, muscle activation, and movement patterns synchronously based on the comprehensive approach.

Since DKV is a kind of sensorimotor dysfunction resulting in maladaptation in muscle activation and movement pattern, the CCEP can be applied to improve this condition as well [14, 24].

On the other hand, previous studies on the effectiveness of exercise program interventions to improve DKV are open to criticism for a number of reasons. First, although they have recommended many different neuromuscular training programs such as stabilization [28], balance [29], and plyometric training [30], they have failed to pay due attention to DKV-related changes over distal and proximal segments as the result of chain reactions [26]. Second, these studies have reported conflicting findings on desirable intervention effects [31–36]. Hence, the current study aimed to explore the effectiveness of the CCEP on lower extremity kinematics and the strength of the selected muscles in participants with DKV. We mainly intended to develop and evaluate preventive approaches for all individuals prone to pain and musculoskeletal injuries due to impaired movement patterns, such as DKV.

## Methods

### Study design

This study was a parallel-group randomized wait-list controlled trial conducted at the Health and Sports Medicine Department University of Tehran, Tehran, Iran. The Ethics Committee on Research obtained ethical approval (Ethic code: IR.UT.REC.1395024). The protocol has been approved in the Registry of Clinical Trials (Registration N: IRCT20180821040843N1) on 30/12/2018. Before the study, all participants read and signed their informed consent forms.

### Participants

A sample size of 26 participants was calculated using G*Power software (version3.1.9.2; Kiel, Germany) based on the desired power of 80%, alpha of 0.05, and effect size of 0.7 reported in previous studies investigating kinematic variables ranging from 0.6 to 0.88 [37, 38]. 15 participants were included for each group to consider possible dropouts.

A total of 90 asymptomatic volunteers without a history of regular physical exercise were assessed using the screening tests for eligibility for enrollment in the study. Following the warm-up, they performed five sequential single-leg squat tasks to approximately 60° of knee flexion.

Participants between the ages of 18 and 28 years were recruited if the midpoint of the patella moved medially to the great toe during the single-leg squat, at least three out of five trials [39–41]. The primary examiner both visually observed and recorded the trials using a digital camera for more accurate scoring [39]. Test-retest reliability for this test was higher than 0.90 in a pilot study**.** Participants were excluded if they had a history of lower extremity injury restricting movement [42, 43], any visible musculoskeletal deformities in lower extremities and upper quarter in normal standing posture [44, 45], a bodyweight beyond normal range, i.e. BMI < 18 or BMI > 28, or if they lost more than three sessions, or two sequential sessions of the intervention program [46].

### Randomization

**Fig. 1:**
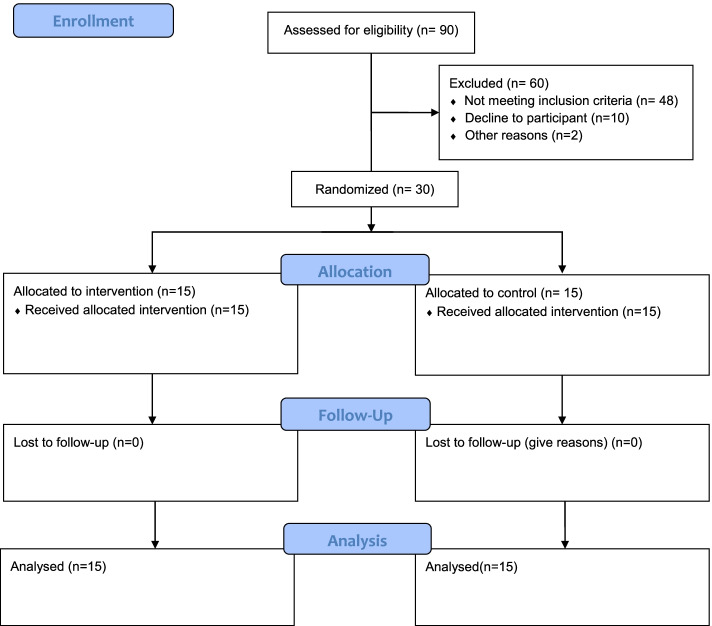
CONSORT flow chart

A total of 30 volunteers, illustrated in the CONSORT diagram (Fig. 1), met all the inclusion criteria randomly assigned to the intervention and control groups. (Intervention: Age: 24.5 ± 2.5 years, height: 178.9 ± 7.2 cm, and weight: 76.7 ± 2.5 kg. control: Age: 24.4 ± 1.5 years, height: 179.8 ± 3.5 cm, and weight: 74.7 ± 3.9 kg). Computer-generated randomization was used in a 1:1 ratio, followed by a masked allocation by opening the sequentially numbered, checkmate, and secured envelopes. A card inside each envelope indicated the group where the participant was randomly allocated, i.e. intervention or control groups.

### Intervention (CCEP)

The intervention commenced after one week of baseline assessments. Participants in the intervention group attended three sessions per week for eight weeks which is deemed as a reasonable duration for both neuromuscular and physiological adaptations [47], with at least a 48-hour recovery period between sessions [47, 48]. After completing the study, the control group also received the intervention program due to ethical considerations. The CCEP was planned in initial, improvement, and maintenance phases. The load of exercises progressed during these phases provided that the exercise program was appropriately performed from the examiner’s point of view.

**Fig. 2:**
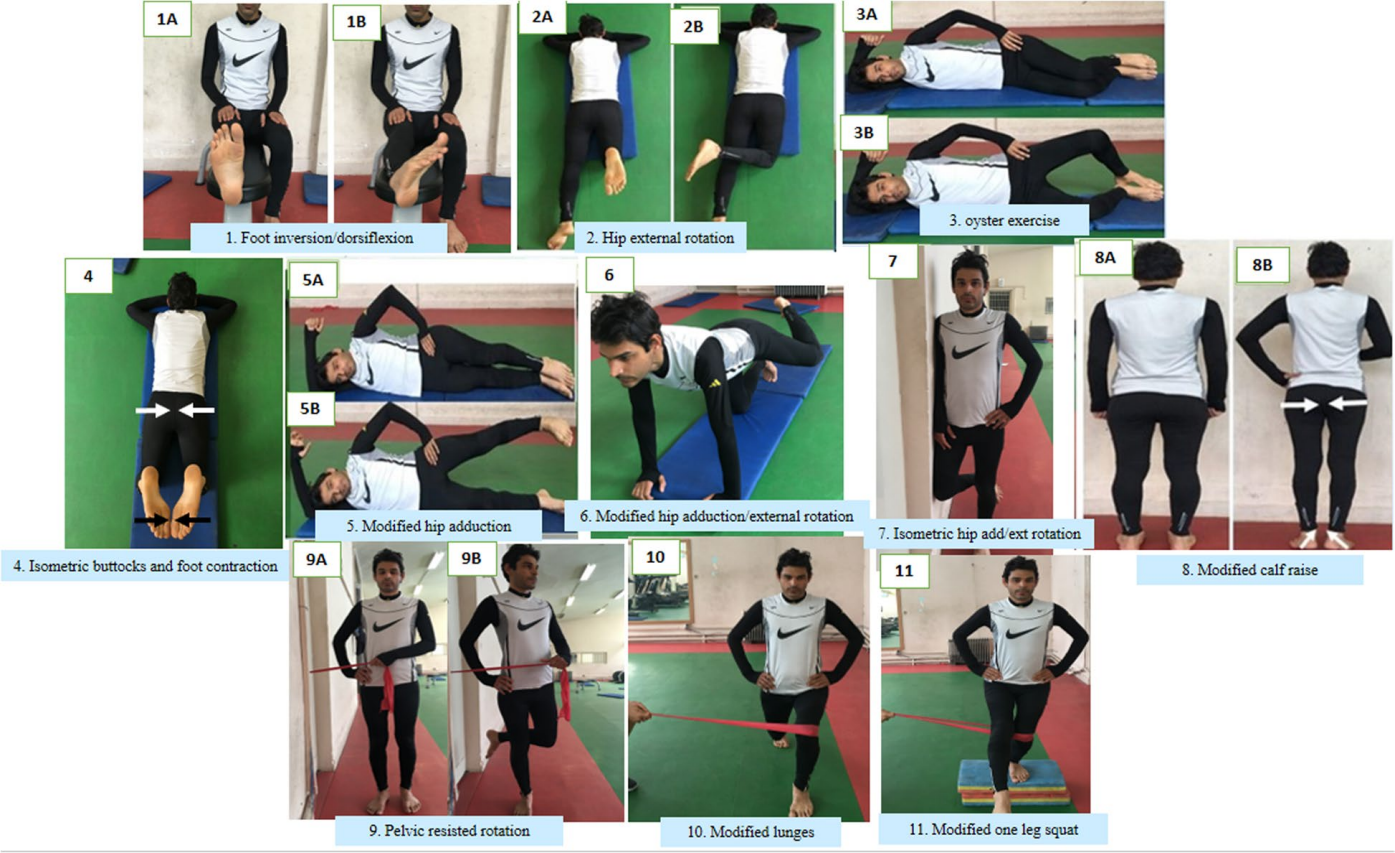
Comprehensive Corrective Exercises Program (CCEP)

According to Lederman’s rehabilitation pyramids [49], the researcher intended to improve the sensorimotor abilities (parametric and primary proprioceptive abilities) in the initial phase. He passively positioned each participant into the appropriate alignment by giving necessary feedback and helping them to contract the inhibited muscles isometrically, focusing mainly on improving their cognition and quality of performing the exercises. Participants completed exercises 1–3 illustrated in Fig. 2 in non-weight-bearing positions.

Next, the progression of exercise load occurred during the improvement phase (3–6 weeks) using the exercises 4–8 shown in Fig. 2 to produce necessary tissue adaptations [50, 51]. Participants were supposed to improve their synergistic abilities, such as co-contraction and reciprocal activation of the muscles, in different weight-bearing positions progressing from side-lying to quadruped, sitting, and ultimately different standing positions. The progression of exercise repetitions and intensity was due only if participants could display high-quality performance of exercises. Finally, participants were supposed to maintain the training and developmental adaptations in the maintenance phase (7–8 weeks) consisting of exercises 9–11 shown in Fig. 2 [51]. During this phase, exercises progressed functionally to provide an adequate challenge for sensorimotor adaptation. It was highly emphasized that participants maintained the proper alignment of the involved segment and all other body segments during exercises and functional movement patterns. Besides, different kinds of visual, oral, and tactile feedback were provided. Indeed, the researcher continuously provided verbal cues on proper alignment in their performance. If necessary, the researcher would have modeled the exercise movements or used a mirror so that participants could find out their mistakes or even touched and directed them how to perform the exercises accurately.

The exercise program has been summarized in Table 1.

**Table 1:** Exercise program

Session (in week)	week							
	1	2	3	4	5	6	7	8
1	1,2,3	1,2,3	4,5,6,7,8	4,5,6,7,8	4,5,6,7,8	4,5,6,7,8	4,5,6,7,8,9,10,11	4,5,6,7,8,9,10,11
2	1,2,3	1,2,3	4,5,6,7,8	4,5,6,7,8	4,5,6,7,8	4,5,6,7,8	4,5,6,7,8,9,10,11	4,5,6,7,8,9,10,11
3	1,2,3	1,2,3	4,5,6,7,8	4,5,6,7,8	4,5,6,7,8	4,5,6,7,8	4,5,6,7,8,9,10,11	4,5,6,7,8,9,10,11

### Data collection

Data were collected in the biomechanics laboratory of the University of Tehran. Fourteen reflective markers were placed on anatomical landmarks according to the Plug-in Gait method: ASISs, PSISs, lateral side of tights, lateral epicondyles of the knee, lateral side of shanks, lateral malleolus, and center of the calcaneus. Participants were instructed to perform single-leg squat trials to approximately 60° of knee flexion while maintaining a rhythm of 2 seconds for both ascending and descending phases. Each participant stood on the dominant leg, with the toes straight forward, the non-weight-bearing leg was in the position used in the screening test (90° knee flexion and 45° hip flexion), and hands were crossed to opposite shoulders. Then, they were asked to perform three sequential trials of the single-leg squat approximately to 60° of knee flexion. The lower extremity kinematic data were recorded at maximum knee flexion angle for each participant and the average of three trials was calculated. The acceptable range of the knee flexion angle for each trial was from 50° to 70°. The test-retest reliabilities for all kinematics measures were above 0.80 in a pilot study. Three-dimensional trajectory data for DKV were acquired using a 6-camera motion analysis system (Vicon; Oxford Metrics LTD, Oxford, UK). Trajectory data were digitally recorded using Nexus software (Windows Version 2.6.1) and sampled at 240 Hz. The Butterworth filter filtered kinematic data using a low-pass, zero lag, and fourth-order with a 10-Hz cut-off frequency to obtain smooth and coordinated data [52]. ISB recommendation on definitions of joint coordinate systems was followed for kinematic variable measurements [53].

### Hip dynamometry procedures

The isometric strength of interested muscles was measured by Manual Muscle Test System (Model 01163, Lafayette ICA). Three measurement trials with a 30-second rest between three trials were performed, and participants were asked to complete the movement exerting maximum strength. The average of these three trials was recorded as each participant’s performance in each test [11]. Hip abductor strength was measured by side-lying on a treatment table while the back was against the wall, and the top leg was in touch with the wall when the underneath leg was flexed, and the testing leg was straight and resting on a pillow. Resistance band was fastened against these muscles approximately 2 cm proximal to the lateral femoral condyle [11]. The dynamometer was stabilized with straps to eliminate the potential examiner strength bias [54]. For measuring external hip rotation, participants were seated while both knee and hip were flexed at 90°. Resistance band was used approximately 2 cm proximal to ankle lateral malleolus [55]. For a more accurate comparison of the two groups, the muscle strength values were normalized to body weight.

Test-retest reliabilities of muscle strength measurement for the external hip rotator and abductor strength performed were 0.89–0.96 in a pilot study, respectively.

Testing lower extremity kinematics and hip strength was performed one week after the intervention. Pre and post-data were obtained by the same examiners blinded to the study groups.

### Statistical analyses

Data were analyzed with descriptive and inferential statistics using IBM SPSS 20 (*p* ≤ 0.05). Shapiro–Wilk test was used to ensure the normality of data’s and Levene’s test was also employed for testing the homogeneity of variances. One-way ANOVA was used to detect differences between the baseline demographic characteristics of the groups in pre-test. Moreover, one-way ANCOVA using pre-test as the covariate was used to investigate the differences between the intervention and control groups at the end of the 8-week CCEP. Effect sizes were calculated using partial eta-squared (η2) interpreted as small (0.01), medium (0.09), and large (0.14) [56]. In addition, the minimal clinically important difference (MCID), defined as the minimum change in a treatment outcome, was calculated based on the following formula: MCID = SD × 0/5 [57].

## Results

All 30 participants completed the exercise programs, and their data were included in data analysis. No statistically significant differences were found in the baseline demographic characteristics of the groups and dependent variables (*P* > 0.05).

As per Table 2, ANOCVA results revealed significant effects of the 8-week CCEP. There were significant improvements in all kinematics variables in the intervention group in comparison to the control group in post-test for knee varus/valgus (F = 24.31, *p* = 0.001, η^2^ = 0.43), femur adduction/abduction (F = 6.93, *p* = 0.01, η^2^ = 0.23), femur medial/lateral rotation (F = 10.50, *p* = 0.004, η^2^ = 0.33), tibial rotation (F = 10.53, *p* = 0.005, η^2^ = 0.27), hip abductor strength (F = 19.90, *p* = 0.001, η^2^ = 0.49), and hip external rotator strength (F = 25.73, *p* = 0.001, η^2^ = 0.56).

**Table 2:** The results of ANCOVA for between-group differences

variable	Intervention group		Within- group mean differences	ANCOVA			Control group		Within- group mean differences	between-group mean differences	%95 CI	
	Pre-test	Post-test		F	P^†^	η^2^	Pre-test	Post-test				
											lower	upper
Knee varus (+)/valgus (−) (degree)	−19.71 ± 7.48	− 11.24 ± 3.6	−8.47	24.31	0.001^*^	0.43	−18.51 ± 7.15	−19.84 ± 7.42	− 1.33	−8.24	5.15	12.68
Femur adduction (+)/ abduction (−) (degree)	11.28 ± 4.98	4.38 ± 6.65	6.9	6.93	0.01^*^	0.23	11.07 ± 8.71	9.98 ± 8.33	1.09	−5.6	−10.31	−1.21
Femur medial(+)/lateral rotation(−) (degree)	14.23 ± 4.0	7.46 ± 4.88	6.77	10.50	0.004^*^	0.33	17.48 ± 8.29	16.75 ± 8.02	0.73	−9.29	5.26	11.72
Tibial medial (+)/lateral rotation (−) (degree)	−23.79 ± 3.56	−17.23 ± 4.66	−6.56	10.53	0.005^*^	0.27	−23.13 ± 7.23	− 23.94 ± 6.28	−0.81	−6.71	−20.31	−14.07
Abductor strength)newton(	0.35 ± 0.02	0.37 ± 0.02	−0.02	19.90	0.001^*^	0.49	0.35 ± 0.04	0.34 ± 0.04	0.01	0.03	0.01	0.03
External rotator strength)newton(	0.21 ± 0.02	0.24 ± 0.02	−0.03	25.73	0.001^*^	0.56	0.21 ± 0.03	0.22 ± 0.03	−0.01	0.02	0.01	0.04

†: P represents differences in post-training between intervention and control groups.

*: Indicates significant differences *P* < 0.05

The results of the MCID calculation indicated that the amount of difference observed in the effect of training protocol on research variables was higher than the values required for the minimum clinical difference. In other words, the differences observed in the study are clinically significant as well (Table 3).

**Table 3:** The results of the MCID

	Minimum required changes calculated by the formula	The amount of observed changes
Knee varus (+)/valgus(−)	1. 8	8. 47
Femur adduction (+)/ abduction(−)	3. 32	4. 2
Femur medial(+)/lateral rotation(−)	2. 44	9. 77
Tibial medial(−)/lateral rotation(−)	2. 33	6. 47
Abductor strength	0.01	0. 2
External rotator strength	0.01	0.3

## Discussion

This study aimed to investigate the effect of the eight-week CCEP on the kinematics and strength of lower extremities in males with dynamic knee valgus. The results supported the primary hypothesis on the statistically and clinically significant effectiveness of the CCEP on the kinematics and muscle strength of lower extremities in males with DKV.

A number of previous studies have indicated significant improvement in muscle strength using exercise programs [11, 20]. However, there are not any reports in terms of DKV and related lower extremity kinematics following the related interventions [11, 21, 58], which is attributed to mere focusing on strength training based upon structural approach. The present study is one of the pioneers in showing significant improvements in DKV and related kinematics with large effect sizes (0.14 < η2) which may be due to targeting all distal and proximal contributors to DKV using a comprehensive intervention attending alignment, muscle activation, and movement pattern simultaneously.

It is hypothesized that exercises targeting hip muscle strength can fine-tune knee kinematics [59], which is supported by some studies [29, 54]. However, evaluating the effectiveness of functional motor control exercises and isolated hip abductor strengthening on knee kinematics, Palmar et al. (2015) reported significant improvement in both groups’ hip abductor strength, but not in knee kinematics. Interestingly, strength improvement in the isolated hip abductor strengthening group was greater than the functional motor the control group, but kinematics improvement in the functional control group was more than the other group [11]. It should be noted that paying mere attention to muscle strength or length while ignoring other involved factors, as in structural approach, cannot improve knee kinematics measures. Next, although strength improvement has a remarkable role in improving DKV, correction of motor control is even more crucial.

The current research used exercises 1–3 illustrated in Fig. 2 to improve both strength and cognition in the early intervention phase which is essential to modify or facilitate motor behavior and control [49]. Therefore, participants in the CCEP group that contracted underactive targeted muscles in the initial phase using an internal focus of attention could achieve satisfactory ability in both contracting and utilizing these muscles. Then, the improvement phase was devoted to developing synergistic abilities using exercise 4–8 shown in Fig. 2. Finally, in the maintenance phase, participants practiced functional patterns using exercises 9–11 shown in Fig. 2 to provide an adequate challenge for sensorimotor adaptation and neuromuscular development, or rather utmost motor control improvement. This may account for the significant outcome and reported large effect sizes in lower extremity kinematics and muscle strength in the intervention group.

Most of the previous studies failing to improve lower extremity kinematics have been more hip dominant in terms of muscle groups being targeted using strength training in participants with DKV [11, 20, 21]. Some studies have identified that weak plantar-flexion strength and subsequent foot pronation reduce ankle range of motion and increase internal tibial rotation leading to compensations in the proximal segment, and thus the more valgus position of the knee during the descent phase of the squat [1, 38]. Accordingly, a crucial contributing factor to DKV that has been ignored in most studies is a deficit in plantar flexor performance. In the present study using the comprehensive approach, distal contributing factors were also considered throughout all exercises. Therefore, it can be regarded as one of the pioneers attending both ankle and hip segments simultaneously for treating DKV, which may account for significant improvements in knee kinematics with large effect sizes in the intervention group.

Moreover, the impaired movement pattern is associated with the reorganization of the cerebral cortex [60]. Therefore, retraining muscle activation patterns by using neuromuscular function and motor learning principles through applying movement education and different kinds of feedback can restore proper muscle unit recruitment patterns [49, 61]. Notably, some studies have displayed that utilizing any type of feedback and movement education as part of the intervention can influence the lower extremity kinematics [62, 63]. Accordingly, proper performance of exercises in all phases and provision of various types of feedback and movement education may justify improvement in participants’ movement patterns [64], and thus lower extremity kinematics and muscle strength, respectively.

Finally, the results of the MCID calculation indicated that the amount of difference observed in the effect of training protocol on research variables was higher than the values required for the minimum clinical difference. In other words, the observed changes were not only statistically, but also clinically significant after applying the CCEP which verifies the effectiveness of this program in the present study.

There are some limitations in the present study. Some studies reported the critical role of ankle plantar/dorsiflexion range of motion on DKV [1, 38], while the current study is limited to measure the same, which can be associated with a portion of improvement in the kinematics variable. Second, since the efficacy of the CCEP was examined on asymptomatic non-athlete participants’ DKV, the findings may not be generalizable to athletes. Future studies should be conducted to investigate the effectiveness of the CCEP on DKV related kinematics and muscle strength in at-risk athletes. Finally, the present study was performed only on male participants due to the impossibility of access to female participants.

## Conclusion

The CCEP proved effective for significant improvement in the selected variables of lower extremity kinematics and muscle strength in participants with DKV highlighting the importance of adopting a comprehensive approach for improving DKV. In other words, practitioners need to consider proximal and distal segments simultaneously for improving DKV using the comprehensive approach.

## Data Availability

The datasets used and/or analyzed during the current study available from the corresponding author on reasonable request.

## Abbreviations

DKV: Dynamic knee valgus
CCEP: Comprehensive corrective exercise program
